# The apelinergic system as an alternative to catecholamines in low-output septic shock

**DOI:** 10.1186/s13054-018-1942-z

**Published:** 2018-01-19

**Authors:** David Coquerel, Xavier Sainsily, Lauralyne Dumont, Philippe Sarret, Éric Marsault, Mannix Auger-Messier, Olivier Lesur

**Affiliations:** 1 Division of Intensive Care Units, Department of Medicine, Université de Sherbrooke, 3001 - 12e Avenue Nord, Sherbrooke, QC J1H 5 N4 Canada; 2 Division of Cardiology, Department of Medicine, Université de Sherbrooke, Sherbrooke, Québec Canada; 3 Department of Pharmacology-Physiology, Université de Sherbrooke, Sherbrooke, Québec Canada; 4 Pharmacology Institute of Sherbrooke, Faculty of Medecine and Health Sciences, Université de Sherbrooke, Sherbrooke, Québec Canada

**Keywords:** Apelinergic system, Apelin (APJ) receptor, Biased signaling, Decatecholaminization, Inodilator, Septic shock

## Abstract

Catecholamines, in concert with fluid resuscitation, have long been recommended in the management of septic shock. However, not all patients respond positively and controversy surrounding the efficacy-to-safety profile of catecholamines has emerged, trending toward decatecholaminization. Contextually, it is time to re-examine the “maintaining blood pressure” paradigm by identifying safer and life-saving alternatives. We put in perspective the emerging and growing knowledge on a promising alternative avenue: the apelinergic system. This target exhibits invaluable pleiotropic properties, including inodilator activity, cardio-renal protection, and control of fluid homeostasis. Taken together, its effects are expected to be greatly beneficial for patients in septic shock.

Septic shock is a life-threatening condition initiated by an acute systemic inflammation with unbalanced host responses to microbial infection [1]. Persistent hypotension related to generalized vasodilation, refractory constrictive responsiveness, huge plasma capillary leak syndrome, coagulation/fibrinolysis imbalance, and metabolic disturbance highlighted by elevated bloodstream lactates are hallmarks of worst outcome in this critical condition [2]. Indeed, an alarming multiple organ failure (MOF) occurrence, aggravated by sustained low blood pressure/perfusion, is closely associated with high morbidity, long-term sequelae, and elevated mortality in septic shock [3].

Preventing insufficient blood delivery to tissues is rapidly recommended as a first-line treatment of septic shock after time-limited fluid resuscitation [2, 4]. This mandates the administration of catecholamine vasopressors such as norepinephrine (NE), a predominantly selective α-adrenergic receptor (α-AR) agonist [4]. Importantly, hemodynamics bedside assessment reveals a high prevalence of myocardial dysfunction which can be detected early in up to 60% of septic patients [5]. In such cases, when cardiac output remains low despite fluid resuscitation (defined per se as a low-output septic shock), additional stimulation of the β-adrenergic receptor (β-AR) by administration of dobutamine is suggested as a strong positive ino-/chronotrope drug [4]. However, whether recommended or only attempted, refractoriness of cardiac response to dobutamine is common in low-output septic shock, which negatively affects treatment efficacy and critically impacts survival, with increased mortality rates of up to 90% [6, 7]. Thus, can we do better than dobutamine? Yes, indeed, provided that lessons taught by history are remembered [8].

In this respect, alternative therapies with better potency/efficacy, less undesired effects, and improved vaso-/cardio-protective impact are urgently needed. The endogenous apelinergic system has recently emerged as a compelling target to sustain cardiovascular function in shock. Indeed, cardiac contractility and vascular tone, fluid homeostasis and kidney function, as well as energy metabolism, inflammatory response, and thrombosis are all physiological hallmarks impacted by apelin receptor (APJ) engagement (Fig. 1) [9–12]. Collectively, the above effects would be beneficial in septic shock and related conditions, with potential added value for outcome. Considering the proven “druggability” of G-protein-coupled receptors (GPCRs) and existing preclinical data, we outline the promising therapeutic potential of the apelinergic system in critically ill septic patients, with a focus on the stabilization of failing cardiovascular hemodynamics and kidney function.

**Fig. 1:**
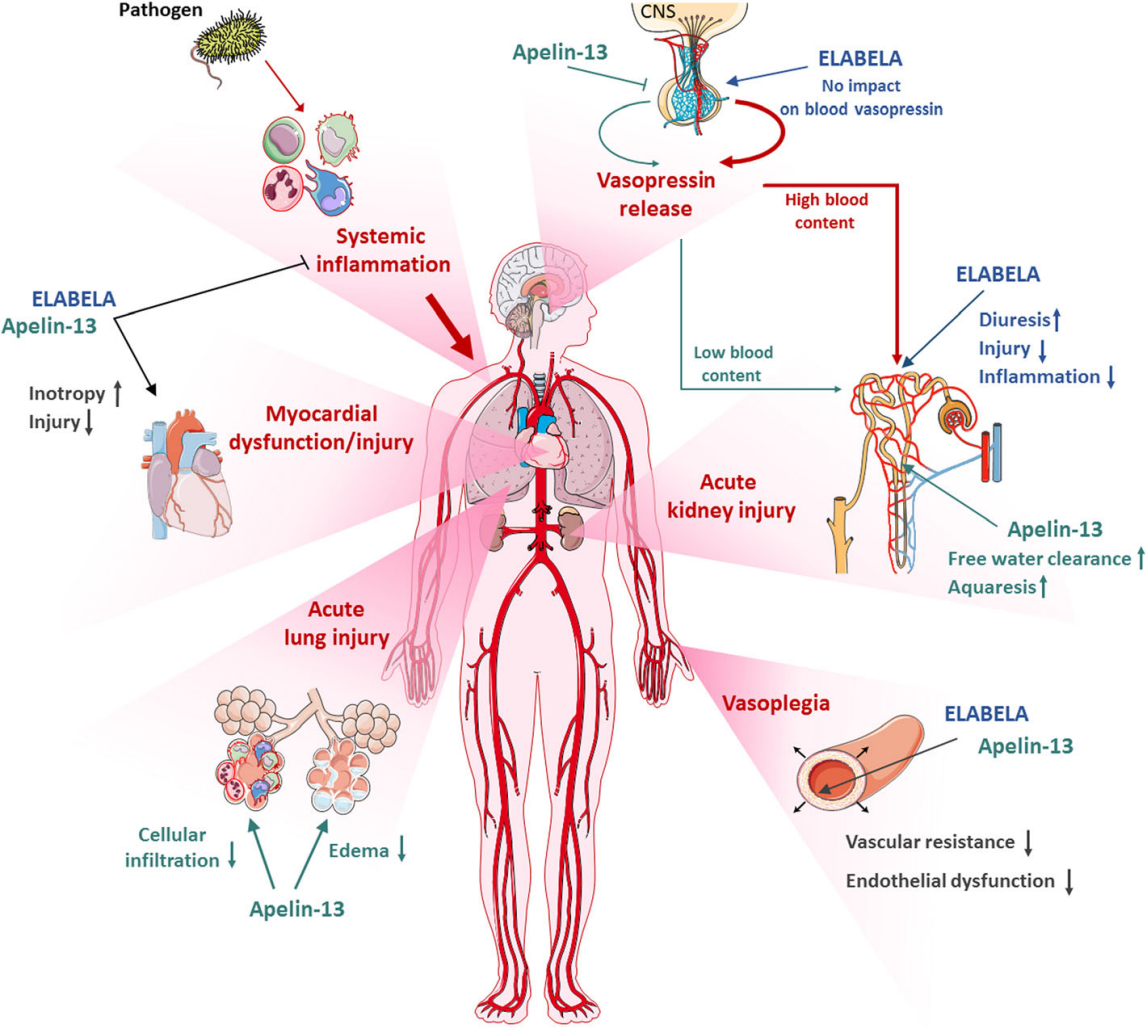
Potential impacts of modulating the apelinergic system in human septic shock with multi-organ failure (MOF). Acute and continuous infusions of the endogenous apelin receptor (APJ) ligands Apelin-13 (APLN-13) and Eleabela (ELA) display several beneficial effects in preclinical septic shock (i.e., endotoxin and cecal ligation and puncture models) as well as in sepsis-related organ failure. Both APLN-13 and ELA reduce bloodstream and tissue inflammation, improve cardiovascular hemodynamics (e.g., enhanced inotropy, reduced pre- and after-load, as well as vascular permeability) and enhance diuresis. Specifically, APLN-13 and ELA exhibit a differential interplay with the vasopressinergic system and therefore modulate fluid homeostasis. APLN-13 alleviates pituitary AVP release, thus inducing low blood AVP and enhanced aquaresis. In contrast, ELA stimulates diuresis in a pressure- and kidney-dependent manner without modified blood AVP, preserving functional water reabsorption and contributing to enhanced plasma volume. Both APLN-13 and ELA infusions confer tissue protection and contribute to reduced mortality and improved outcomes in experimental septic shock. Inhibition of platelet function has been recently described as a novel property of APLN-13, potentially relevant to septic shock, but not addressed in this perspective. *CNS* central nervous system

## The need for decatecholaminization of critically ill patients

**Fig. 2:**
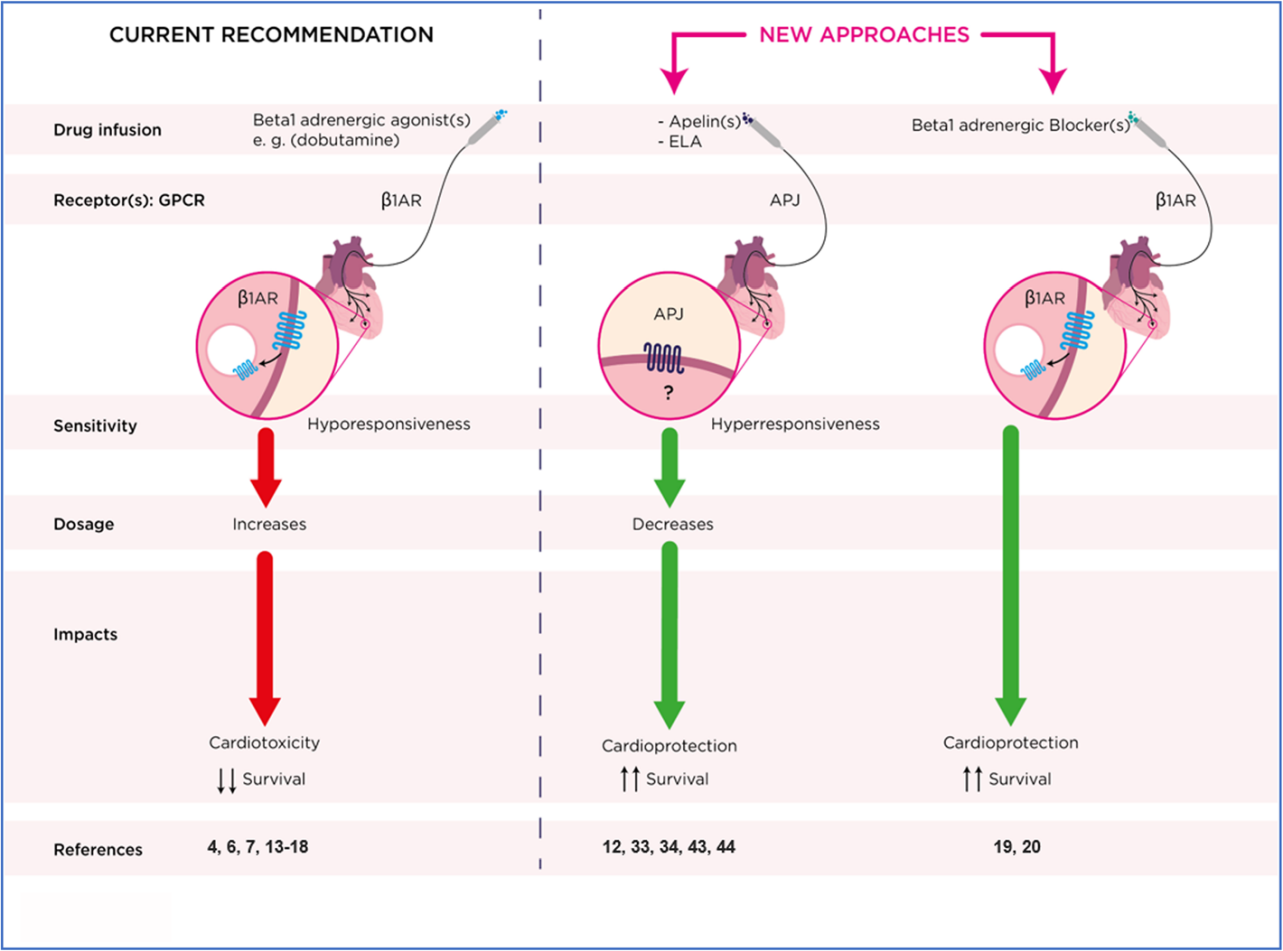
Hemodynamic drug support in septic shock-related myocardial dysfunction: pharmacological state-of-the-art evidence and new approach concepts. Current recommendations after optimal fluid resuscitation are the introduction of adrenergic agonists with emphasis on the β1AR agonist dobutamine when cardiac index remains low, and somewhat often a hyporesponsiveness/increased dosage profile along with β1AR myocardial down-regulation. In spite of this decreased AR availability, cardioprotection and improved survival have been obtained with β1AR blockade. Apelinergic agonists offer cardio-protection and improved outcomes with a high responsiveness/dosage profile at the pre-clinical side. References supporting assumptions are cited in the text. *ELA* Eleabela

Although useful vaso-/cardio-active agents to manage septic shock, catecholamines increase oxidative stress, interfere with cellular energy metabolism, disturb immunological response, and thus undermine their therapeutic value, sometimes exacerbating pre-existing myocardial dysfunction in sepsis [6, 13, 14]. Moreover, important endogenous fractions of mostly inefficient/oxidized circulating catecholamines combined with desensitized β-AR contribute to the hyporesponsiveness of cardiovascular sympathetic activation in septic shock [15–17]. Consequential excessive exogenous catecholamines (including dobutamine) are used to increase cardiac index, often without tangible benefits yet with exacerbated side effects and worse outcomes [18]. Interestingly, despite almost half a century of dobutamine use, β-blocking the septic heart is now proposed for cardioprotection, with recent evidence of the feasibility and effectiveness at both preclinical and clinical levels [19, 20]. Thus, rather than searching for a catecholamine with the best pharmacological properties, a new paradigm called “decatecholaminization” is proposed to partially or completely spare exogenous catecholamine use [21, 22]. Nonetheless, administering β-blockers is not the only way to achieve cardiovascular protection, and other non-adrenergic pathways hold great potential toward such a goal (Fig. 2).

In this context, GPCRs are attractive targets for the development of new drugs with beneficial cardiovascular effects on sepsis. For instance, engagement of the vascular arginine vasopressin (AVP) receptor V_1A_ represents a potential target in septic shock [23]. Indeed, low-dose AVP infusion is helpful to restore vascular tone, with catecholamine-sparing ability in septic patients, and additional beneficial effects have been observed with selepressin (a selective V_1A_ receptor agonist) in both a relevant preclinical model of sepsis and septic shock patients [24, 25]. Targeting the angiotensin system could also be beneficial in patients with NE-refractory shock, by acting on the angiotensin II (Ang II) type 1 receptor (AT_1_ receptor), another GPCR with strong vasopressor activity [26]. However, AVP, Ang II, and all their derivatives are pure vasopressors without obvious direct supportive impact on the failing heart but result in potential inappropriate increased myocardial workload and, moreover, have significant drawbacks in unselected patients. With these considerations in mind, our group recently hypothesized that the inodilator properties (i.e., positive inotropic and vasodilator effects) of the apelinergic system might offer superior therapeutic value, compared to the standard of care, for the treatment of low-output septic shock. By combining those aforementioned capacities, apelinergic agonists would improve arterial–ventricular couplings and overall cardiac index delivery as well as organ perfusion.

## Functional and protective cardiovascular impacts of the apelinergic system

An unknown receptor sharing high sequence homology with the angiotensin II type 1 receptor (AT_1_) gene was identified in 1993 and denominated APJ (Apelin Peptide Jejunum) [27]. This novel receptor, not activated by Ang II, remained an “orphan” GPCR until 1998, when a peptide isolated from bovine stomach homogenates was identified as a selective ligand [28]. APJ is expressed by a wide range of eukaryotic cells and is prevalent in the central nervous system and peripheral organs, including lung, heart, vasculature, and kidney [29]. APJ activation is essentially triggered by two distinct endogenous peptides, i) apelins (APLNs) and ii) the recently discovered (in 2013) ELABELA (ELA; also known as Toddler or Apela) (Fig. 2). APLNs are members of the neuropeptide/adipokine families, and are physiological regulators of cardiovascular function, fluid homeostasis, and energy metabolism [9, 10]. Mature endogenous APLNs—apelin-36, apelin-17, and apelin-13 (APLN-13)—possess overlapping bioactive properties, but APLN-13 is the dominant isoform detected in human heart, vessels, and bloodstream [30, 31]. Physiologically, APLNs increase cardiac contractility in isolated hearts with equivalent efficiency to isoproterenol but with a much more sustained action through activation of protein kinase Cɛ and extracellular signal-regulated kinase 1/2 signaling pathways [32]. Additionally, myosin long-chain kinase activation represents a downstream mechanism by which APLNs could sensitize cardiac myofilaments to Ca^2+^ [30]. Increased cardiomyocyte cytosolic pH and intracellular Ca^2+^ content through sodium hydrogen (NHE) and sodium calcium (NCX) exchanger activation have also been suggested to be involved in APLN-dependent stimulation of cardiomyocyte contractility [33]. Lastly, APLN-dependent dromotropic effects through sodium channel (INa) activation may potentially confer antiarrhythmic abilities [34]. In vivo hemodynamic impacts of APLNs (e.g., induced nitric oxide-dependent and -independent decline in systemic vascular resistance and after load; increased load-independent myocardial contractility and enhanced cardiac index and stroke volume) were confirmed both in preclinical models [35, 36] and at the bedside in humans [37]. Thus, APLNs are clearly members of the “inodilator” family.

ELA, on the other hand, is a long peptide that also activates APJ with similar binding affinity to APLNs [38]. First identified in a “noncoding” DNA region and then ignored, ELA is a potent regulator of early cardiac development in zebrafish [39]. ELA is highly expressed in undifferentiated human embryonic stem cells [40] and kidney [41], and has restricted expression in endothelial cells of adult human vessels [42]. Very recently, an early ELA defect was closely linked to hypertension-related preeclampsia [43]. Beyond its role in development, the ELA/APJ signaling axis is effective in promoting angiogenesis and counteracting Ang II production in adult rodents [44].

Recent results from our group and others demonstrated that exogenous delivery of APLN-13 is beneficial in experimental models of sepsis (e.g., endotoxemia, peritonitis, burns), reducing MOF and improving outcomes [12, 45, 46]. In fact, circulating and myocardial APLN-13 levels are low or weakly reactive in experimental sepsis and septic shock patients [47]. Compared to dobutamine continuous infusion, APLN-13 significantly increases survival and robustly improves left ventricular performance with reduced inflammation and stress [45]. Furthermore, APJ blockade exacerbates myocardial dysfunction and mortality in endotoxin-challenged rats, suggesting that the endogenous apelin system does contribute to counteracting life-threatening hemodynamic alterations [45]. ELA is also powerful and stronger than APLN-13 in stimulating healthy isolated hearts and improving hemodynamics in rats with peritonitis. This superior in vivo inotropic impact of ELA over APLN-13 was confirmed by others [41], including in right hearts with pulmonary arterial hypertension (PAH) [42]. In addition, continuous ELA infusion drives beneficial outcomes over APLN-13 and optimizes pressure–volume relationships of the Frank–Starling curve in a volume-dependent manner [12]. This later observation seems attributable to a differential interplay between APLN-13 and ELA with the vasopressinergic system in the regulation of kidney water reabsorption. Reduction of circulating levels of pro-inflammatory cytokines induced by APLN-13 and ELA also contributes, reducing myocardial injury and systemic vascular permeability, with preserved plasma volume and hemodynamics [12]. Importantly, while sepsis dampens myocardial responsiveness to β1AR agonists, the apelinergic potency on the cardiac response is boosted under systemic inflammatory conditions [45] or polymicrobial infection [12], thus increasing its potential as a therapeutic target (Fig. 2).

## The apelinergic system in fluid homeostasis and renal failure

Body fluid homeostasis is another important physiological role of the apelinergic system that would be beneficial in the management of the septic condition [9]. This system regulates both diuresis and thirst [48], and both APLN-13 and ELA stimulate urinary output and water intake [12]. Conversely, an abnormal fluid balance was observed in APJ-null mice [49]. APLN-13 enhances urinary output through direct and specific vasodilation of efferent renal arterioles, and the expression of APJ in collecting ducts further suggests aquaretic functions, reflecting an APLN-driven specific interplay with the vasopressinergic system [50]. Indeed, AVP binding to V_2_ receptors activates Gα_s_-mediated adenylyl cyclase activity and leads to aquaporin 2 (AQP2) apical docking, driving water reabsorption and decreased diuresis. Activation of APJ coupled to Gα_i_ counterbalances the action of AVP, preventing AQP2 membrane re-localization through adenylyl-cyclase inhibition [50]. APLNs also act in the central nervous system, inhibiting AVP neuron activity and consequent pituitary release in the bloodstream, with increasing aquaresis [48].

During experimental sepsis, whereas continuous infusion of APLN-13 or ELA both restore hemodynamics, only ELA is effective in reducing kidney dysfunction/injury and significantly improves fluid homeostasis, thus limiting hypovolemia [12]. Indeed, APLN-13 infusion lowers blood AVP levels in rats with peritonitis and tilts urinary balance toward undesired aquaresis with subsequent plasma volume loss. Conversely, ELA, which overall shares APLNs’ physiological effects, does not modify blood AVP content and improves fluid balance through preserved AVP-dependent renal water reabsorption. Moreover, ELA reduces sepsis-induced kidney acute injury and inflammation compared to APLN-13 [12]. Such observation is consistent with the exhaustion of AVP release observed in human sepsis, which is suspected to promote sustained hemodynamic failure and immune dysfunction [51, 52]. Indeed, AVP supply affords a non-catecholaminergic pathway to limit hypotension in patients with septic shock [23]. These findings indicate that ELA and APLN-13 differentially affect the cardio–renal axis, mainly through opposing effects on vasopressinergic system counter-regulation, especially on an AVP-driven pathway, which could be centrally mediated and possibly related to the existence of an alternative cell-surface receptor yet to be discovered [40], or to distinct cellular outcomes of ELA binding to APJ. 

This opens a wider discussion on the opportunity to optimize biased apelinergic agonists by modulating their structure–signaling relationship, with the final objective of selecting molecules specific for individualized clinical phenotypes.

## Potential of biased apelinergic agonists to improve hemodynamic support in septic shock

**Fig. 3:**
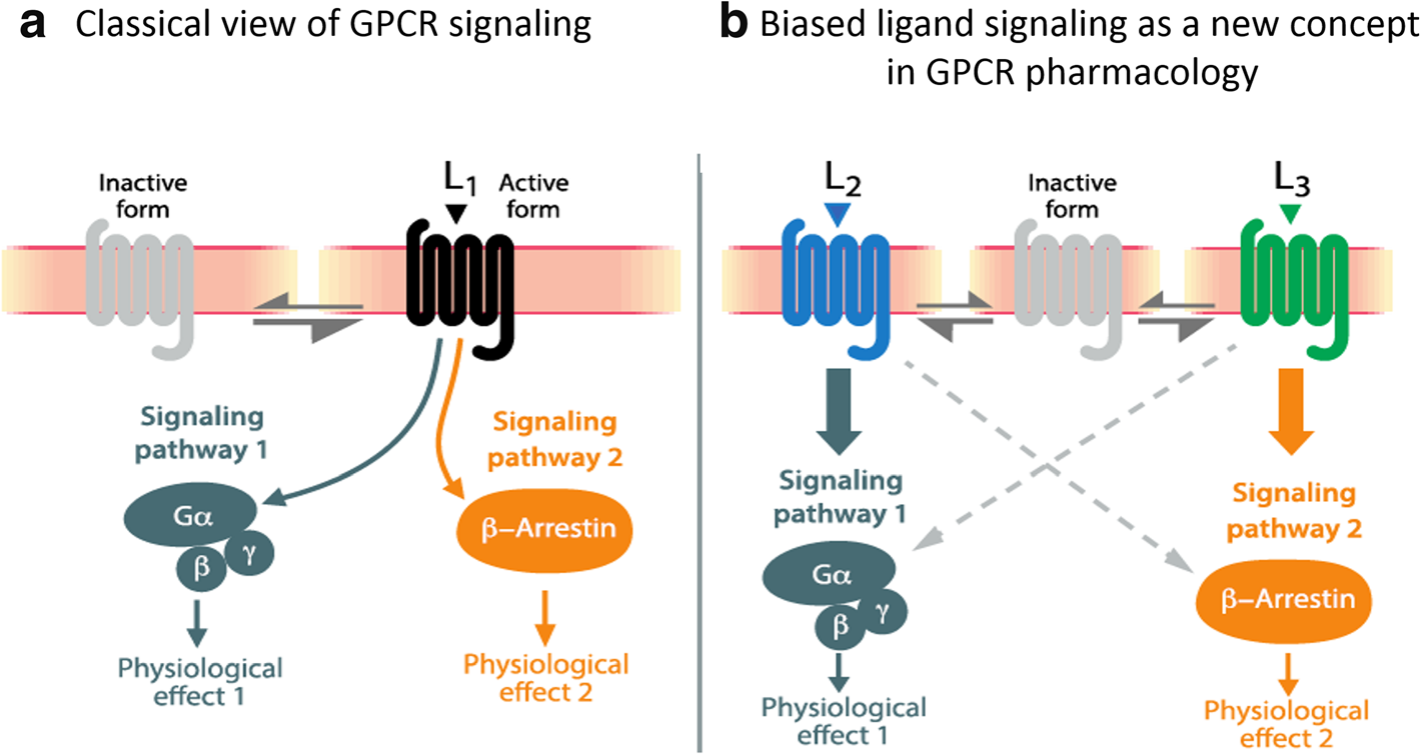
The concept of biased signaling. **a**) In the classic model, ligand L1 binds and elicits a set of signaling pathways leading indiscriminately to multiple physiological effects. **b**) In biased signaling, ligand L2 biases the receptor toward signaling pathway 1, whereas ligand L3 induces bias for pathway 2, leading to distinct physiological outcomes

During the past decade, advances in APLN research have revealed a ligand-dependent physiological response, suggesting several signaling pathways downstream of APJ. Traditionally, GPCR activation was considered to stimulate indiscriminately both G protein-dependent and -independent pathways, irrespective of ligand structure (Fig. 3) [53]. However, some ligands can selectively activate favorable pathways and/or block the contribution of undesirable ones, leading to the concept of biased signaling. Fundamentally, this concept (i.e., functional selectivity) adds an additional layer of complexity to our understanding of the pharmacological action of drugs targeting GPCRs (Fig. 3) [54]. It can be harnessed advantageously, through drug design and structural modifications, to primarily trigger desired cellular outcomes in the hope of significantly improving risk/benefit ratios in pathophysiological conditions. Indeed, previous studies have already demonstrated the value of targeting the apelinergic system in cardiovascular processes such as heart failure, leading to the discovery of biased APJ agonists [55].

According to current knowledge, canonical APJ signaling involves Gα_i_ activation after stimulation by APLNs, leading to inhibition of adenylyl cyclase, decreased intracellular cAMP, and subsequent physiological effects [28]. As mentioned above, a specific interplay between natural apelinergic agonists (e.g., APLN-13 vs ELA) and the vasopressinergic system could induce distinct APJ/Gα_i_-driven outcomes on fluid balance and renal or cardiac functions during experimental sepsis. APJ activation also commits scaffolding proteins β-arrestins, which typically promote receptor internalization and initiate G-protein-independent signaling cascades [56]. Interestingly, β-arrestin signaling has beneficial properties with regard to inhibiting sepsis-related inflammatory response [57]. Furthermore, β-arrestin overexpression dramatically attenuates sepsis-induced myocardial dysfunction [58].

Consistent with these experimental data, our group is currently elucidating the structure–activity relationship of APLNs and ELA, providing insight into binding and signaling to discover novel APJ biased ligands [38]. Overall, biased compounds are critical tools to improve the understanding of APJ-mediated cellular processes and their physiological consequences. Ultimately, those with advantageous effect(s) or devoid of undesirable effects and associated with specific signaling pathways can be selected and trialed in sepsis or any other indication potentially associated with GPCR targets. To date, the jury is still out and investigations to associate APJ signaling with in vivo phenotypic cardiovascular and renal functions is a conundrum that will have to be solved with the help of biased agonists demonstrating beneficial therapeutic impacts and reduced undesired activities in sepsis.

## Conclusions

After its “first-in human” pilot study, the apelinergic system recently gained credibility at both the preclinical and early clinical stages as a potential therapeutic in chronic heart failure. Although exhibiting enriched pleiotropic abilities, recent evidence supports its protective impact on the cardiovascular and renal axes, which are prevalently compromised in septic shock with an acute myocardial dysfunction. Further knowledge and the development of dedicated biased agonists are ongoing and mandatory to bring to the market a new family of apelinergic drugs able to substitute for catecholamines in the treatment of sepsis.

## Abbreviations

α-AR: Alpha-adrenergic receptor
β-AR: Beta-adrenergic receptor
Ang II: Angiotensin II
APJ: Apelin peptide jejunum (apelin receptor)
APLN: Apelin
AQP2: Aquaporin 2
AT1 receptor: Angiotensin II type 1 receptor
AVP: Arginine vasopressin
cAMP: Cyclic adenosine monophosphate
ELA: ELABELA
GPCR: G-protein coupled receptor
Ina: Sodium channel
MOF: Multiple organ failure
NE: Norepinephrine
NHE: Sodium hydrogen exchanger
NCX: Sodium calcium exchanger
PAH: Pulmonary arterial hypertension
